# Effect of model race and viewing perspective on body attractiveness and body size assessment in young Caucasian women: an eye-tracking study

**DOI:** 10.1007/s00426-018-1138-9

**Published:** 2018-12-15

**Authors:** Victoria Rodway, Bethany Tatham, Kun Guo

**Affiliations:** School of Psychology, University of Lincoln, Brayford Pool, Lincoln, LN6 7TS UK

## Abstract

Research has indicated that Caucasian women gaze more often at waist–hip and chest regions than other local body areas when assessing female body attractiveness and body size, and this stereotypical gaze distribution is further modulated by their own body satisfaction and body composition. However, little is known whether the model race and viewing perspective could affect women’s body-viewing gaze behaviour and body perception. Here, we presented female body images of Caucasian, Asian and African avatars in a continuum of common dress sizes in full frontal, mid-profile and rear view, and asked young Caucasian women to rate the perceived body attractiveness and body size. Their body-viewing gaze distributions were then correlated with their behavioural responses, their own body composition and body satisfaction. Our analysis revealed a clear in-group favouritism, in which Caucasian women tended to rate Caucasian avatars more attractive and slimmer than Asian and African avatars. Their body-viewing gaze patterns, on the other hand, were not affected by avatar race but were modulated by viewing perspectives. The frontal-view body (especially upper-body and waist–hip regions) attracted the highest proportion of viewing time, followed by the mid-profile view and then the rear-view body. Furthermore, our participants’ own body composition and satisfaction level did not affect their judgement of other women’s body attractiveness and body size, but could influence their gaze allocation at local body features. It seems that both body perception and body-viewing gaze behaviour are subject to group and individual biases.

## Introduction

It is well acknowledged that female body attractiveness and body size assessments play important roles in women’s social behaviour, mental and physical health (e.g. eating disorder). Previous research has indicated those factors related to women’s health and fertility, such as body mass index (BMI), body fat and waist-to-hip ratio (WHR), are correlated with body attractiveness and size judgements (Singh, 1993; Tovée, Reinhardt, Emery, & Cornelissen, 1998; Weeden & Sabini, 2005; Singh & Singh, 2011). For instance, slender figures with low WHR and large breasts are often rated as more attractive (Singh & Young, 1995). Consequently, the waist–hip and chest regions tend to transmit diagnostic cues for body attractiveness and size judgements, and attract more frequent visual inspection than other local body features from young female viewers in the tasks of free viewing, body attractiveness and body size judgements (Cornelissen, Hancock, Kiviniemi, George, & Tovée, 2009; Hall, Hogue, & Guo, 2011, 2014; Cundall & Guo, 2017).

Interestingly, when viewing other female bodies with high attractiveness rating and preferred body size, women’s gaze allocation is modulated by their own body satisfaction and body composition (Cundall & Guo, 2017). It has been reported that women with low own body satisfaction scores tended to engage more in body comparison with the others (measured via Physical Appearance Comparison Scale, PACS) to evaluate their own body appearance, and this internal-driven comparison process could be manifested in their body-viewing gaze allocation (Cundall & Guo, 2017). They often looked less at the body regions rated high in own-body satisfaction, but looked more at those regions rated low in own-body satisfaction, implying satisfaction might reduce the need for comparison of those body parts which they felt confident about (Jansen, Nederkoorn, & Mulkens, 2005; Cundall & Guo, 2017). Taken together, this individualised gaze comparison process and stereotypical pattern of gaze concentration at the waist–hip and chest regions indicate that body-viewing gaze behaviour in young women might be driven by the need for social comparison to establish or improve one’s own attractiveness (Hahn & Perrett, 2014), as suggested by the mate selection theory which postulates women judge their own attractiveness relative to other women to assess their own likelihood for successful mate selection or to monitor potential attractive competitors posing a greater threat to partner sexual fidelity (Pawlowski & Dunbar, 1999; Hughes, Harrison, & Gallup, 2004; O’Connor & Feinberg, 2012).

However, there are two limiting factors which may affect the generalisation of these research findings on female body perception and associated viewing behaviour. The first one is race effect. The typical design in previous research is to examine women’s body perception in viewing of female body images of the same racial group (e.g. Hall et al., 2011, 2014; Cundall & Guo, 2017). However, the idealised female body shape and WHR alter across cultures and are subject to societal influence. For instance, women with a lower BMI (~ 20 to 22) and around 0.7 WHR are perceived as the most attractive in Western culture (Tovée, Edmonds, & Vuong, 2012), whereas heavier bodies are preferred in non-Western culture (e.g. 0.8 WHR for African and 0.9 WHR for native south Americans) (Sugiyama, 2004; Tovée, Swami, Furnham, & Mangalparsad, 2006; Reeve, Kelly, & Welling, 2016). It is, therefore, unclear to what extent the observed Caucasian body attractiveness judgement and body-viewing gaze behaviour in Caucasian viewers could be extended to view female bodies of other races. Given our current multi-culture society and the existence of clear differences in body shape and body composition across racial groups (e.g. when compared with Caucasian, Africans tend to have longer legs but shorter torsos, whilst Asian typically show the opposite pattern; Seeman, 1998), it would be interesting and valid to examine whether Caucasian women would use the same cognitive strategy to assess female bodies of different races. Previous research has shown that own-race individuals are considered more familiar, resulting in an in-group favouritism (Zebrowitz, Bronstad, & Lee, 2007). Therefore, by adding race as a factor, we could examine whether a race-invariant mental representation of body attractiveness (including diagnostic bodily cues for attractiveness judgement) would exist in young Caucasian women, and whether the social comparison process, a potential mechanism underlying female body perception, differs according to the race of female body being assessed.

The second limiting factor is the viewing perspective. Previous body perception studies commonly use female body images in full frontal view. Naturalistic body perception, however, involves different viewing perspectives that can change the visibility of local body features and reveal the body shape in varying details (e.g. rear view would provide more accurate assessment about hip size than frontal view). As recent research has noticed that exploring the same face from different viewing angles could quantitatively influence facial expression judgement (e.g. perceived expression intensity) and gaze allocation at local facial features (Guo & Shaw, 2015), it is plausible that varying viewpoints may modify gaze distribution in body viewing and subsequently influence body attractiveness and body size judgements.

Clearly, research on body perception using images of women of different racial groups in multiple viewing perspectives would have higher ecological validity. Hence, this eye-tracking study was designed to systematically address the identified research limitations in female body perception. To mimic real world situations, we presented high-resolution body images of well-controlled Caucasian, Asian and African avatars in a continuum of common dress sizes in full frontal, mid-profile and rear view, and asked young healthy Caucasian female viewers to rate the perceived body attractiveness and body size. Their gaze distributions in body viewing were then correlated with their behavioural ratings, their own body composition measurements (BMI, WHR and chest size) and regional body satisfaction ratings. Guided by previous findings, we hypothesised that (1) participants would attend to waist–hip and chest regions to assess body attractiveness and body size regardless of model race (Cundall & Guo, 2017); (2) partipcants would show rating preference for Caucasian models due to in-group favouritism (Zebrowitz et al., 2007); (3) body-viewing gaze allocation at a given body feature would vary across viewpoints, similar to those reported in face-viewing gaze behaviour (Guo & Shaw, 2015); (4) participants’ own body composition, regional body dissatisfaction and their tendency for social and body comparisons (measured via PACS) would increase their gaze allocation to the concerned body regions (Cundall & Guo, 2017).

## Materials and methods

### Participants

Advertising through the university subject pool, 36 Caucasian female undergraduate students, aged between 19 and 23 years (20.14 ± 0.17, mean ± SEM), were recruited to participate in this study in return for course credit. All participants reported sexual orientation (31 heterosexual, 3 bisexual and 2 homosexual), no history of eating disorders and had normal or corrected-to-normal visual acuity. Prior to the study, the research purpose, experimental tasks and procedure had been explained to the participants, and written informed consent was obtained from each of them. The Ethical Committee in School of Psychology, University of Lincoln, approved this study (PSY171858), and all procedures complied with the British Psychological Society Code of Ethics and Conduct.

### Body images

**Fig. 1:**
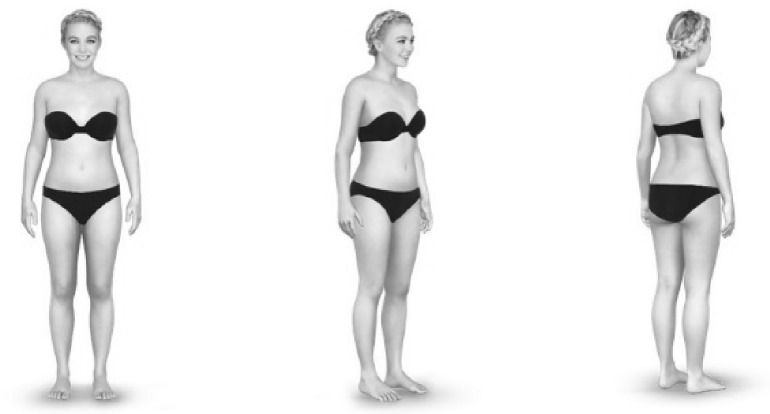
Body image example of a female Caucasian avatar in UK dress size 10

Full-colour high-resolution female body images dressed in plain black underwear (computer-generated avatars) were created via a free online virtual fitting room (http://www.trymetail.com). Measurements of typical UK dress sizes commonly found in high street stores (obtained from http://www.asos.com) were entered into the software to produce full body images depicting seven dress sizes ranging from UK6 to UK18 (size 6, 8, 10, 12, 14, 16 and 18; height and inner leg measurements were standardised at 165 and 74 cm, respectively; breast cup sizes ranged from AA to C and increased in parallel to dress size). To make the body images more representative of the general population, for each dress size, nine avatars were created to represent three different races (Caucasian, Asian and African) and three different cup sizes. Each avatar had similar age, the same hairstyle and similar facial expression with no distinctive facial or body markings. In total, 63 images (7 dress sizes × 3 races × 3 cup sizes) were created for the testing. Each image showed the same avatar from three different viewing perspectives: frontal view, 45° mid-profile view, and 135° rear-profile view (i.e. the three viewing perspectives were presented simultaneously; see Fig. 1 for an example). The size of the images was set to 900 × 450 pixels (34.62° × 17.31°).

The digitized body images were presented through a ViSaGe graphics system (Cambridge Research Systems, UK) and displayed on a non-interlaced gamma-corrected colour monitor (30 cd/m^2^ background luminance, 100 Hz frame rate, Mitsubishi Diamond Pro2070SB) with the resolution of 1024 × 768 pixels. At a viewing distance of 57 cm, the monitor subtended a visual angle of 40° × 30°.

### Procedure

During the eye-tracking experiment, the participants sat in a chair with their head restrained by a chin rest and viewed the display binocularly. Horizontal and vertical eye positions from the dominant eye (determined through the hole-in-card test) were measured using a pupil-centre/cornea-reflection Video Eyetracker Toolbox with 250 Hz sampling frequency and up to 0.25° accuracy (Cambridge Research Systems, UK). Eye movement signals were first calibrated by instructing the participant to follow a fixation point (FP, 0.3° diameter, 15 cd/m^2^ luminance) displayed randomly at one of nine positions (3 × 3 matrix) across the monitor (distance between adjacent FP positions was 10°).

After the calibration procedure, the participants pressed the response box to initiate a trial. The trial was started with an FP randomly displayed 10° left or right to the screen centre to minimize central fixation bias and possible spatial attention bias to the left visual field. If the participant maintained fixation for 1 s, the FP disappeared and a testing image was presented at the centre of the screen for 5 s. The participants were instructed to “rate body attractiveness and body size as accurately as possible”, and verbally report the perceived body attractiveness rating on a 9-point scale (1 represents ‘not attractive at all’ and 9 represents ‘extremely attractive’) and body size rating on a scale ranging from UK size 6–18. During the testing no feedback was given, and each body image was displayed once in a random order.

Considering that own-body satisfaction measures might temporarily enhance own-body awareness and consequently affect body-viewing gaze behaviour, the body satisfaction measures were conducted after the eye-tracking task to avoid potential carryover effects. Participants were required to complete three questionnaires which included the following. (1) Body composition: participants’ weight, height, waist and hip sizes were measured to calculate BMI (weight/height^2^) and WHR. Participants’ actual bra size, actual UK dress size, ideal (self-preferred) bra size, ideal dress size and ideal waist and hip sizes were also recorded through self-report measures. (2) Body satisfaction: participants self-rated their satisfaction with each of six body regions (face, breasts, waist, hip, arms and legs) on a 9-point scale, 1 being the most dissatisfied and 9 being the most satisfied. (3) Physical Appearance Comparison Scale (PACS; Thompson, Heinberg, & Tantleff, 1991): PACS is a five-item scale used to measure an individual’s tendency to use social comparison to evaluate their own appearance. It includes items such as “In social situations, I sometimes compare my figure to the figures of other people” and responses range from never (1) to always (5).

### Data analysis

All the collected data were analysed off-line. For eye movement data, the software developed in Matlab computed horizontal and vertical eye displacement signals as a function of time to determine eye velocity and position. Fixation locations were then extracted from raw eye-tracking data using velocity (less than 0.2° eye displacement at a velocity of less than 20°/s) and duration (greater than 50 ms) criteria (Guo, Mahmoodi, Robertson, &Young, 2006). To determine gaze allocation within key body regions (Hall et al., 2011; Cundall & Guo, 2017), each body was divided into five regions of interest: face (including hair), upper body (from the base of the neck to the end of the rib cage), waist–hip region (including stomach, hips, and pubic region), arms (including hands) and legs (including feet). The viewing time allocated to each region was normalised in proportion to total viewing time sampled in that trial.

A series of repeated measures analysis of variance (ANOVAs) was conducted to examine the effect of avatar race and dress size on participants’ body attractiveness and body size judgements, and the effect of viewing perspective and avatar race on their body-viewing gaze allocation. For each ANOVA, Greenhouse–Geisser correction was applied where sphericity was violated, and a Bonferroni adjustment was made for post hoc multiple comparisons.

## Results

### Effect of avatar race and dress size on body attractiveness and body size judgements

**Fig. 2:**
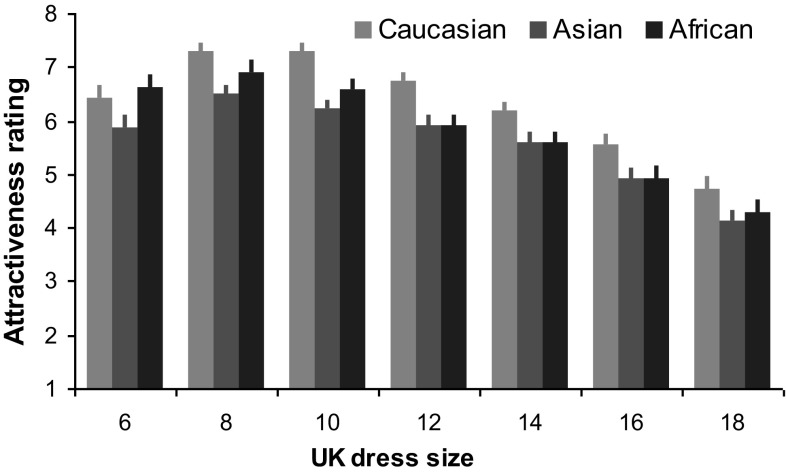
Attractiveness ratings for each avatar race in each dress size. Error bars represent SEM

Body attractiveness judgement: to explore to what extent body attractiveness judgements were affected by avatar race and dress size, a 3 (race) × 7 (dress size) ANOVA was conducted with attractiveness rating as the dependent variable. The analysis revealed significant main effect of avatar race (*F*(1.69, 59.13) = 17.22, *p* < 0.001, $\eta _{{\text{p}}}^{2}$ = 0.33; Fig. 2) with Caucasian receiving the highest attractiveness rating (Caucasian vs Asian, *p* < 0.001; Caucasian vs African, *p* = 0.007) and Asian and African receiving indistinguishable ratings (*p* = 0.08), and significant main effect of dress size (*F*(1.95, 68.15) = 68.59, *p* < 0.001, $\eta _{{\text{p}}}^{2}$ = 0.66) with size 8/10 and 18 rated as the most and least attractive, respectively, and larger sizes (14–18) rated less attractive than smaller sizes (6–12, all *p*s < 0.05). The significant race × dress size interaction (*F*(5.35, 187.17) = 5.09, *p* < 0.001, $\eta _{{\text{p}}}^{2}$ = 0.13) further demonstrated that the most attractive dress size was a size 8 or 10 for Caucasian (size 8: 7.29 ± 0.17, size 10: 7.31 ± 0.14, *p* = 1.00) and Asian avatars (size 8: 6.5 ± 0.17, size 10: 6.22 ± 0.14, *p* = 0.76), but a size 6 (6.6 ± 0.23), 8 (6.91 ± 0.22) or 10 (6.58 ± 0.21) for African avatars (size 6 vs size 8, *p* = 0.29; size 6 vs size 10, *p* = 1.00; size 8 vs size 10, *p* = 0.25), whereas the least attractive dress size was a size 18 for all avatar races (Caucasian 4.72 ± 0.24, Asian 4.13 ± 0.21, African 4.29 ± 0.24).

**Fig. 3:**
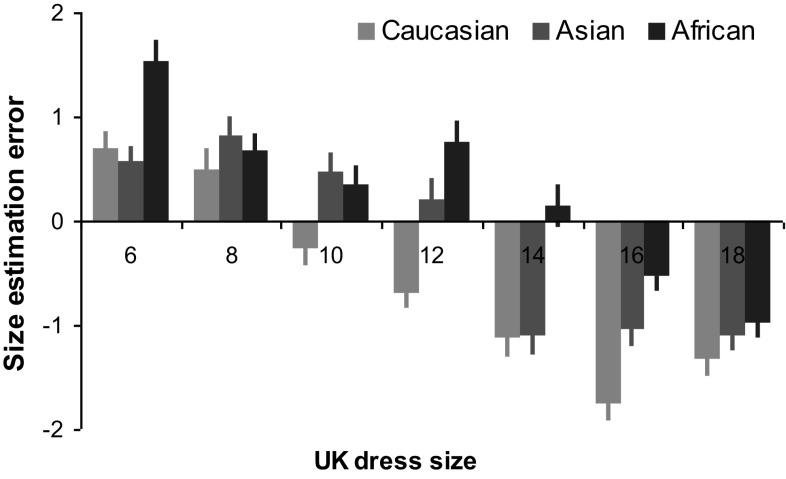
Mean body size estimation error between reported and actual size for each dress size and avatar race. Error bars represent SEM

Body size judgement: to explore whether body size judgements were affected by avatar race and dress size, a 3 (race) × 7 (dress size) ANOVA was conducted with body size estimation error for each dress size (reported size − actual dress size) as the dependent variable. The analysis revealed significant main effect of avatar race (*F*(2, 70) = 74.52, *p* < 0.001, $\eta _{{\text{p}}}^{2}$ = 0.68; Fig. 3) and dress size (*F*(3.45, 120.85) = 77.00, *p* < 0.001, $\eta _{{\text{p}}}^{2}$ = 0.69), and significant interaction effect (*F*(7.62, 266.64) = 9.57, *p* < 0.001, $\eta _{{\text{p}}}^{2}$ = 0.22). Generally, participants tended to overestimate for smaller dress sizes (6–12) and underestimate for larger dress sizes (14–18; all *p*s < 0.05). The magnitudes of their estimation errors were systematically correlated with the changes of actual avatar dress size (from size 6 to 18, the mean estimation errors were 0.94 ± 0.13, 0.66 ± 0.15, 0.19 ± 0.16, 0.09 ± 0.16, − 0.69 ± 0.16, − 1.11 ± 0.13, and − 1.14 ± 0.14 respectively; *r* = − 0.98, *p* < 0.001). Furthermore, the directions of these estimation errors (overestimation vs underestimation) were avatar race dependent, especially for sizes 10–14. For example, participants underestimated dress size if avatars were a Caucasian size 10 (− 0.23 ± 0.17) or 12 (− 0.69 ± 0.16), yet overestimated dress size if they were a size 10 or 12 Asian and African avatars; whereas for size 14, they would underestimate both Caucasian and Asian avatars. Analysis of linear regression further indicated that the averaged break points between overestimation and underestimation for Caucasian, Asian and African avatars were size 9.23, 11.07 and 13.53, respectively.

### Effect of viewing perspective and avatar race on body-viewing gaze allocation

**Fig. 4:**
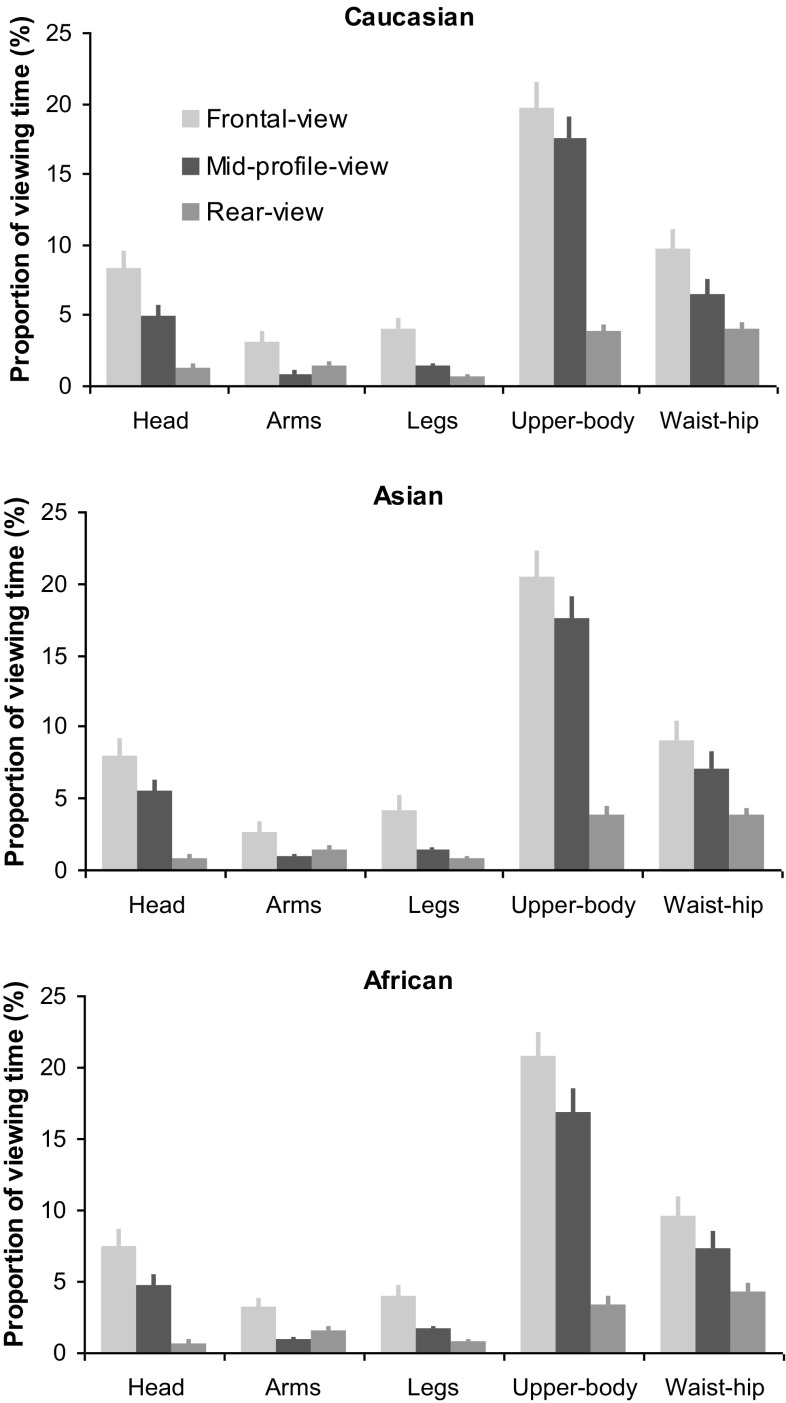
Average proportion of viewing time directed at individual body regions in each avatar race presented at frontal, mid-profile and rear-profile view. Error bars represent SEM

To further examine how gaze allocation at each body region altered across viewing perspectives and avatar races, a 3 (viewing perspective) × 3 (race) × 5 (body region) ANOVA was conducted. The analysis revealed significant main effect of viewing perspective (*F*(1.23, 43.08) = 67.21, *p* < 0.001, $\eta _{{\text{p}}}^{2}$ = 0.66; Fig. 4) with the frontal-view avatar attracting the largest amount of viewing time (44.47% ± 2.09), followed by the mid-profile view (31.54% ± 2.10) and then the rear-view avatar (10.75% ± 0.74) (frontal vs mid-profile view, *p* = 0.007; frontal vs rear view, *p* < 0.001; frontal vs rear view, *p* < 0.001), and significant main effect of body region (*F*(2.34, 81.73) = 49.97, *p* < 0.001, $\eta _{{\text{p}}}^{2}$ = 0.59) with the upper-body region attracting the highest proportion of viewing time followed by the waist–hip region and the head, whereas both the legs and arms received the least but indistinguishable proportion of viewing time (upper-body vs other body regions, all *ps* < 0.001; waist–hip vs head, *p* = 0.78; waist–hip or head vs other body regions, *p* < 0.01; legs vs arms, *p* = 1.00; legs or arms vs other body regions, *p* < 0.01). The significant viewing perspective × body region interaction (*F*(3.68, 128.87) = 17.99, *p* < 0.001, $\eta _{{\text{p}}}^{2}$ = 0.34) further revealed that the upper body in the frontal view attracted the largest amount of viewing (20.25% ± 1.78) and the legs in the rear view attracted the least (0.75% ± 0.12). Furthermore, for bodies in both the frontal and mid-profile views, the upper body tended to receive the longest viewing, followed by the waist–hip and head, and then the legs and arms (upper body vs waist–hip/head vs legs/arms, all *p*s < 0.05; waist–hip vs head, *p* > 0.05; legs vs arms, *p* > 0.05); whereas for bodies in the rear view, the upper-body and waist–hip tended to receive similar amount of viewing time (*p* > 0.05) that was also longer than the head, arms and legs (upper body/waist–hip vs head/ arms/legs, all *p*s < 0.05). Additionally, there was no significant race effect (*F*(2, 70) = 0.33, *p* = 0.72, $\eta _{{\text{p}}}^{2}$ = 0.01) or other interaction effects, suggesting avatar race had no clear impact on the body-viewing gaze allocation.

### Individual differences in body perception and associated body-viewing gaze behaviour

A series of correlational analysis were then performed to examine to what extent participants’ own body composition (BMI, WHR and chest size) and their self-rated body satisfaction (body region satisfaction: face, chest, waist, hip, arms, legs and their body ideals) would affect their assessment of other female bodies (attractiveness rating and body size estimation) and associated gaze allocation at each local body region (proportion of viewing time at face, upper body, waist–hip, arms and legs) in each viewing perspective (frontal view, mid-profile view and rear view). As the avatar race did not show any impact on the body-viewing gaze behaviour, participants’ gaze allocation at different avatar races was collapsed together for the correlational analysis.

#### Objective measurements of own body composition

Across our participants, their BMI ranged from 16.7 to 36.7 (23.64 ± 0.69), waist–hip ratio ranged from 0.68 to 1.04 (0.82 ± 0.01), and chest size ranged from cup size A to F with the average of a C cup. Pearson correlations did not reveal any significant association between own body composition (BMI, WHR and chest size) and body assessment of the others (body attractiveness ratings and body size estimations, all *p*s > 0.05), but there was a significant positive correlation between BMI and PACS (*r* = 0.40, *p* = 0.02; Table 1) and a significant negative correlation between WHR and PACS (*r* = − 0.34, *p* = 0.04), suggesting that a higher BMI results in more social comparisons, whereas a smaller WHR results in less comparisons being made. There were also significant negative correlations between BMI and own leg satisfaction scores (*r* = − 0.38, *p* = 0.02; Table 1), between chest size and arms satisfaction scores (*r* = − 0.41, *p* = 0.01), and between chest size and leg satisfaction scores (*r* = − 0.43, *p* = 0.01), suggesting that a larger BMI and chest size tended to result in more dissatisfaction with own arms and legs. Furthermore, when considering their ideal body size (self-reported preferred chest size, waist and hip sizes), there were significant negative correlations between BMI or chest size and all ideal body size indicators (all *p*s < 0.05; Table 1), indicating a preference for a slim body for those with higher BMI and larger chest size. No significant correlation was found between WHR and ideal body size (all *p*s > 0.05).

**Table 1:** Correlation analysis between objective measurements of own body composition and subjective satisfaction of own body composition

		Measured BMI	Measured WHR	Measured chest size
	PACS	0.40 (0.02) *	− 0.34 (0.04) *	0.31 (0.06)
Subjective satisfaction	Face	0.18 (0.29)	− 0.07 (0.71)	− 0.17 (0.32)
	Chest	0.26 (0.13)	0.17 (0.32)	0.32 (0.06)
	Waist	− 0.24 (0.17)	− 0.31 (0.07)	− 0.30 (0.07)
	Hips	− 0.32 (0.06)	− 0.08 (0.65)	− 0.20 (0.26)
	Arms	− 0.30 (0.07)	− 0.10 (0.58)	− 0.41 (0.01) *
	Legs	− 0.38 (0.02) *	0.26 (0.13)	− 0.43 (0.01) *
Ideal size	Chest	− 0.55 (0.01) **	0.15 (0.39)	− 0.80 (0.01) **
	Waist	− 0.35 (0.03) *	0.06 (0.75)	− 0.34 (0.04) *
	Hips	− 0.53 (0.01) **	− 0.01 (0.93)	− 0.41 (0.01) *

Note: values in the table represent *r* value (*p* value). *p < 0.05, **p < 0.01

For the associated body-viewing gaze allocation, significant positive correlations were only found between participants’ BMI and viewing time at the upper-body region in mid-profile view (*r* = 0.35, *p* = 0.04; Table 2), and between WHR and viewing time at the upper-body region in mid-profile view (*r* = 0.4, *p* = 0.02). No other significant correlations were observed.

**Table 2:** Correlation analysis between objective measurements of own body composition and viewing time directed at individual body regions at frontal, mid-profile and rear-profile views

	Measured BMI	Measured WHR	Measured chest size
Frontal			
Head	− 0.24 (0.15)	− 0.28 (0.10)	− 0.08 (0.66)
Upper body	− 0.10 (0.56)	− 0.01 (0.96)	0.04 (0.81)
Waist–hip	− 0.07 (0.70)	− 0.04 (0.82)	0.17 (0.33)
Arms	0.10 (0.57)	− 0.06 (0.73)	− 0.05 (0.80)
Legs	− 0.02 (0.93)	− 0.16 (0.34)	0.06 (0.72)
Mid-profile			
Head	− 0.16 (0.34)	− 0.23 (0.18)	− 0.10 (0.57)
Upper body	0.35 (0.04)*	0.40 (0.02)*	0.04 (0.81)
Waist–hip	0.14 (0.41)	0.15 (0.39)	0.01 (0.94)
Arms	0.08 (0.66)	0.13 (0.46)	0.03 (0.88)
Legs	− 0.06 (0.73)	− 0.06 (0.74)	0.07 (0.69)
Rear profile			
Head	− 0.22 (0.20)	− 0.16 (0.34)	− 0.16 (0.36)
Upper body	− 0.07 (0.70)	0.28 (0.10)	− 0.17 (0.33)
Waist–hip	− 0.16 (0.37)	0.29 (0.09)	− 0.15 (0.37)
Arms	− 0.20 (0.26)	− 0.03 (0.87)	− 0.14 (0.41)
Legs	− 0.06 (0.75)	− 0.11 (0.52)	− 0.01 (0.94)

Values in the table represent *r* values (*p* value). *p < 0.05, **p < 0.01

#### Subjective satisfaction of own body composition

The participants had an average PACS score of 26.72 ± 1.55 (ranging from 8 to 44) and overall body satisfaction score (across all body regions) of 5.51 ± 0.73. They were most satisfied with their waist (5.89 ± 0.28), followed by face (5.83 ± 0.27), chest (5.64 ± 0.31), legs (5.31 ± 0.31), hips (5.25 ± 0.33) and then arms (5.14 ± 0.29). There was no significant association between PACS or overall own body satisfaction score and body assessment of the others (body attractiveness ratings and body size estimations, all *ps* > 0.05), but there was a significant negative correlation between overall body satisfaction and PACS (*r* = − 0.36, *p* = 0.03), indicating those scoring lower in body satisfaction tended to undertake more frequent appearance comparisons.

For the body-viewing gaze allocation, significant negative correlations were found between PACS and viewing time at the waist–hip region in the rear-view image (*r* = − 0.44, *p* < 0.01; Table 3), and between own leg satisfaction scores and viewing time at the leg region in the frontal-view image (*r* = − 0.45, *p* < 0.01). Furthermore, positive correlations were found between own arm satisfaction and viewing times at the face region in both the frontal-view (*r* = 0.47, *p* < 0.01) and mid-profile view images (*r* = 0.51, *p* < 0.01). No other significant correlations were observed.

**Table 3:** Correlation analysis between subjective satisfaction of own body composition and viewing time directed at individual body regions at frontal, mid-profile and rear-profile views

	PACS	Face	Chest	Waist	Hips	Arms	Legs
Frontal							
Head	− 0.30 (0.08)	0.07 (0.69)	− 0.25 (0.15)	0.21 (0.22)	0.17 (0.32)	0.47 (0.01) **	0.19 (0.27)
Upper body	0.28 (0.10)	− 0.18 (0.30)	0.05 (0.76)	0.07 (0.67)	− 0.21 (0.22)	− 0.22 (0.19)	− 0.10 (0.57)
Waist–hip	− 0.14 (0.41)	− 0.22 (0.20)	0.09 (0.59)	− 0.27 (0.11)	− 0.01 (0.99)	− 0.02 (0.91)	− 0.25 (0.14)
Arms	0.10 (0.56)	0.06 (0.74)	0.01 (0.94)	0.10 (0.57)	0.06 (0.73)	0.07 (0.61)	− 0.17 (0.31)
Legs	0.16 (0.35)	0.08 (0.65)	0.06 (0.71)	0.04 (0.82)	0.15 (0.39)	− 0.12 (0.47)	− 0.45 (0.01)**
Mid-profile							
Head	− 0.29 (0.08)	0.01 (0.99)	− 0.15 (0.38)	0.11 (0.51)	0.12 (0.49)	0.51 (0.01)**	0.17 (0.33)
Upper body	0.12 (0.49)	0.13 (0.45)	0.09 (0.59)	− 0.08 (0.64)	− 0.19 (0.27)	− 0.22 (0.20)	0.24 (0.16)
Waist–hip	0.12 (0.48)	0.31 (0.68)	0.02 (0.93)	− 0.01 (0.98)	0.16 (0.37)	− 0.21 (0.21)	0.03 (0.86)
Arms	− 0.02 (0.92)	− 0.01 (0.99)	0.15 (0.39)	− 0.02 (0.89)	− 0.02 (0.93)	− 0.06 (0.75)	0.05 (0.75)
Legs	0.04 (0.83)	0.27 (0.11)	− 0.03 (0.85)	0.29 (0.08)	0.03 (0.88)	0.03 (0.88)	− 0.28 (0.10)
Rear-profile							
Head	− 0.08 (0.64)	− 0.04 (0.82)	− 0.11 (0.53)	0.03 (0.86)	0.10 (0.55)	0.32 (0.06)	0.15 (0.40)
Upper body	− 0.04 (0.83)	− 0.14 (0.40)	0.17 (0.33)	− 0.04 (0.84)	− 0.24 (0.15)	0.08 (0.65)	0.10 (0.55)
Waist–hip	− 0.44 (0.01)**	0.05 (0.77)	− 0.11 (0.52)	0.06 (0.71)	0.22 (0.19)	0.16 (0.36)	0.09 (0.62)
Arms	0.09 (0.62)	− 0.14 (0.43)	0.24 (0.15)	0.14 (0.42)	− 0.21 (0.22)	− 0.16 (0.34)	0.15 (0.39)
Legs	− 0.09 (0.59)	0.04 (0.83)	0.05 (0.76)	0.19 (0.28)	0.06 (0.72)	0.06 (0.72)	− 0.24 (0.16)

Values in the table represent *r* value (*p* value)

*p < 0.05, **p < 0.01

## Discussion

This study aimed to extend previous research on female body perception by specifically examining how the model race and viewing perspective could affect body attractiveness and body size judgements, and associated body-viewing gaze behaviour. Additionally, the effect of individual differences (relating to the measurements of own body composition, body satisfaction and frequency of social comparison) on this body perception process was considered.

Our analysis showed that the avatar race had an evident impact on the body attractiveness and body size judgements. Overall, Caucasian avatars were rated as more attractive (except for size 6) and slimmer (except for size 6 and 8) than Asian and African avatars, suggesting that an in-group favouritism (Zebrowitz et al., 2007) may have caused the preference of familiar, own racial group avatars by our Caucasian participants. The effect of in-group favouritism (e.g. own-race advantages) has also been reported in face identity (Walker & Tanaka, 2003) and facial expression recognition (Elfenbein & Ambady, 2002, 2003), in which human recognition performance is biased (with increased recognition accuracy and shortened reaction time) towards their own as opposed to another race’s faces. Our Caucasian participants, however, did not make more accurate body size judgements for Caucasian avatars than for Asian or African avatars. Perhaps, different cognitive processes are needed for in-group favouritism in face recognition (i.e. categorical judgement) and body perception (i.e. quantity judgement). Nevertheless, our observation in this study implies the possible existence of a template of attractiveness that, for Caucasians, does not match up with the body structure and composition that occur in individuals of Asian and African origin. It should be noted though that the within-subject research design in this study might not fully reveal the magnitude of in-group favouritism in body perception, as our participants might be aware of the social desirability of their responses given the sensitivity of race-related issues in our society. This might have subsequently affected their ratings of body attractiveness and size. It would be interesting to revisit this research question with a between-subject research design.

Irrespective of avatar races, bodies in larger dress sizes (UK 14–18) were consistently rated less attractive than those in smaller ones (UK 6–12), with size 8/10 and 18 being rated as the most and least attractive, respectively. This observation was in agreement with previous research using body images of Caucasian models (Cundall & Guo, 2017). Clearly, although there are anatomical and preferential differences in body shape and composition cross-culturally (such as preferred WHR; Sugiyama, 2004; Tovée et al., 2006), young Caucasian women showed an overall preference for slimmer body size in all tested races, perhaps as a result of thinner female bodies being portrayed as the ideal body shape (particularly) within media platforms (Jiang & Vartanian, 2016). From this perspective, female representation of body attractiveness has an evolutionary foundation, which is arguably reinforced through societal influences.

Interestingly, when judging body size, participants consistently overestimated the smaller dress sizes (UK6, 8), whilst the larger sizes (UK16, 18) were underestimated. This might be partly caused by the participants’ desire of conforming to social etiquette, in which it is inappropriate to call someone skinny or fat (Swami et al., 2008). The cognitive bias in quantative judgements (Hastie & Dawes, 2001), such as an tendency to shift towards the middle of the scale when there is uncertainty in magnitude judgments, might be another contributing factor. It is also possible for the purpose of self-protection, as estimating body sizes closer to own body size would potentially protect self-esteem. In contrast, body size estimations were most accurate for size 10 and 12 avatars, as these sizes are likely to elicit less self-concern about own body size which resulted in more accurate judgements.

Regarding the associated body-viewing gaze behaviour, the avatar race showed no impact on our participants’ body-viewing gaze distribution, indicating their preference for Caucasian avatars was not reflected on the cognitive processing stage of body information selection and extraction. In other words, irrespective of avatar races, the same bodily cues were sampled and analyzed by our participants for assessing body attractiveness and body size. The viewing perspectives, on the other hand, could modify the amount of time directed at the whole body. Across different viewpoints, the frontal-view body tended to attract the highest proportion of viewing time, followed by the mid-profile view and then the rear-view body. It seems that the frontal and mid-profile views are more informative for judging female body shape.

Interestingly, although the viewing time allocated at a given body feature (e.g. waist–hip) was quantitatively different across viewpoints, the overall pattern of gaze distribution at different local body features (e.g. head, upper body, waist–hip, legs and arms) was qualitatively similar across viewpoints (Fig. 4). In particular for both the frontal and mid-profile views, the upper-body region attracted the largest proportion of viewing time, followed by the waist–hip region then the heads, and finally the legs and arms. These findings are consistent with previous observation that both the upper-body and waist–hip regions provide diagnostic cues in assessing sexual maturity, body attractiveness and body size (Cornelissen et al., 2009; Lykins, Ferris, & Graham, 2014; Garza, Heredia, & Cieslicka, 2016).

A reverse pattern of gaze distribution, in which the waist–hip attracted the largest proportion of viewing time followed by the upper body, was observed when the avatars were dressed in full clothes (rather than dressed in underwear in this study) and consequently visual cues from the upper body (e.g. chest size) became more ambiguous (Cundall & Guo, 2017). This would imply that only body features containing clear diagnostic information are likely to receive detailed visual inspection in body viewing and explains why the avatars in this study, who were dressed in underwear, received a large amount of gaze at the upper-body region.

The least amount of visual inspection was directed at the legs and arms, respectively, due to the limited amount of information they provide on body attractiveness and body size perception. In the context of mate selection theory (Buss, 2003), these body features receive little attention from men when looking for a mate (Hall et al., 2011) and thus receive little attention from women when assessing competition and individual mate value.

Regarding individual differences in female body perception, our participants’ own body composition measurements (e.g. BMI, WHR, chest size) and satisfaction level did not affect their judgement of other women’s body attractiveness and body size, but could influence their gaze allocation at local body features. Specifically, individuals with higher BMI and WHR tended to look more at the upper-body region in mid-profile view. As chest is more visible in mid-profile view and consequently its shape and size can be more accurately judged, longer gaze allocation at this region could be for comparative purpose. For example, mate selection theory would suggest that BMI and WHR impact women’s own attractiveness level or ‘market’ value, and the female chest region is indicative of attractiveness (Singh & Young, 1995; Cornelissen et al., 2009). Therefore, women with a larger BMI and WHR (hence with less attractiveness level) view this body region longer as they have a greater need to evaluate other women’s ‘market’ value. Indeed, within our participant group, those with higher BMI and WHR were also more likely to engage in social comparison with other women (indicated by higher PACS scores), possibly due to their dissimilar to the idealised slender frame which is glamorised in Western societies (Smolak & Murnen, 2008). Alternatively, this region attracted more visual attention due to it being less changeable than BMI and WHR. Therefore, gaze allocation was diverted to this neighbouring region (relative to waist–hip) for self-protection purposes (Cundall & Guo, 2017).

Additionally, those individuals scoring higher in PACS attended less often at the waist–hip region in the rear-view bodies, further indicating that the rear view was probably less informative for female body assessment and social comparison than the frontal and mid-profile views. Among self-rated own body feature satisfaction scores, only leg satisfaction score was negatively correlated with viewing at the leg region in the frontal-view bodies, suggesting those participants with high leg satisfaction allocated gaze at alternative body regions. Unlike previous research (e.g. Jansen et al., 2005; Cundall & Guo, 2017), this less frequent own body satisfaction-related gaze comparison or avoidant behaviour (i.e. women look more or less at the body regions rated low in own-body satisfaction) observed in this study may be (at least partly) explained by the clothing effect. Our avatars were dressed in underwear, hence containing little ambiguous information regarding body composition (e.g. waist–hip size) and reducing prolonged viewing and analysis of local bodily cues.

In conclusion, this study has enriched the current research literature by demonstrating an in-group favouritism in body perception, in which body attractiveness and body size judgements are influenced by the viewed body race. The body-viewing gaze allocation, on the other hand, is not affected by the body race but can be modulated by the viewing perspectives. Furthermore, the participants’ own body composition and satisfaction level could influence their gaze allocation at local body features in body viewing. Taken together, it seems that both body perception and body-viewing gaze behaviour are subject to group and individual biases.
